# Prediction and analysis of antifreeze proteins

**DOI:** 10.1016/j.heliyon.2021.e07953

**Published:** 2021-09-08

**Authors:** Ryosuke Miyata, Yoshitaka Moriwaki, Tohru Terada, Kentaro Shimizu

**Affiliations:** Department of Biotechnology, The University of Tokyo, 1-1-1 yayoi, Bunkyo-ku, Tokyo, 113-8657, Japan

**Keywords:** Antifreeze proteins, Prediction, Protein sequences, Amino acids, Random forest

## Abstract

Antifreeze proteins (AFPs) are proteins that protect cellular fluids and body fluids from freezing by inhibiting the nucleation and growth of ice crystals and preventing ice recrystallization, thereby contributing to the maintenance of life in living organisms. They exist in fish, insects, microorganisms, and fungi. However, the number of known AFPs is currently limited, and it is essential to construct a reliable dataset of AFPs and develop a bioinformatics tool to predict AFPs. In this work, we first collected AFPs sequences from UniProtKB considering the reliability of annotations and, based on these datasets, developed a prediction system using random forest. We achieved accuracies of 0.961 and 0.947 for non-redundant sequences with less than 90% and 30% identities and achieved the accuracy of 0.953 for representative sequences for each species. Using the ability of random forest, we identified the sequence features that contributed to the prediction. Some sequence features were common to AFPs from different species. These features include the Cys content, Ala-Ala content, Trp-Gly content, and the amino acids’ distribution related to the disorder propensity. The computer program and the dataset developed in this work are available from the GitHub site: https://github.com/ryomiya/Prediction-and-analysis-of-antifreeze-proteins.

## Highlights

• Construct reliable datasets of antifreeze proteins (AFPs).
• Develop a bioinformatics tool to predict AFPs from their sequences by using random forest.
• Achieved accuracies of 0.961 and 0.947 for non-redundant sequences with less than 90% and 30% identities.
• Using the ability of random forest, dentified the sequence features that contributed to the prediction.

## 1 Introduction

Antifreeze proteins (AFPs) are proteins that protect cellular fluids and body fluids from freezing by inhibiting the nucleation and growth of ice crystals and preventing ice recrystallization, thereby contributing to the maintenance of life in living organisms. They exist in fish, insects, microorganisms, and fungi [1, 2, 3, 4, 5, 6, 7, 8, 9, 10]. Because of their functions, AFPs are expected to be utilized in food processing, cryopreservation of food, tissues, and organs, medicine, and the development of anti-icing materials. Thus, it is imperative to research antifreeze proteins, which are expected to be applied to a vast range of fields.

Previously known AFPs are antifreeze glycoproteins (AFGPs), Type-1 AFP, Type-2 AFP, Type-3 AFP, and Type-4 AFP, which were discovered early in the history of AFP research. AFGPs are proteins with the repeating sequence Ala-Ala-Thr, which is found in polar fishes, and the Thr is modified with β-d-galactosyl-(1,3)-α-n-acetyl-d-galactosamine disaccharide, which is involved in ice crystal binding [11]. Some low-molecular-weight AFGPs have been found to have alanine residues replaced by proline and threonine residues replaced by arginine [12]. Type-1 AFP is an Ala-rich, helix-rich protein found in fish such as flatfish, flounder, and sculpin and is the most widely documented of all AFP types due to its historical background as the first protein structure to be determined [13]. Type-2 AFP is a 14–24 kDa Ca^2+^-dependent, a cysteine-rich globular protein that has been found in fishes such as herring (*Clupea harengus*), cucumber fish (*Osmerus mordax)*, wagtail (*Hypomesus nipponensis), sculpin* (*Hemitripterus americanus*), and whitefish (*Brachyopsis rostratus*) [14, 15, 16]. Type-2 AFP is the largest globular AFP to date homologous to the Ca^2+^-dependent C-type lectin-like domain. Type-3 AFP is a small globular protein with an average molecular weight of about 6.5 kDa with a one-turn alpha-helix and beta-sheet and can be found in Antarctic eelgrass (*Macrozoarces americanus*), Antarctic *eelpout*, and *wolffish* [17]. Type-4 AFP is a new AFP species found in the horned sculpin's plasma, *Myoxocephalus octodecemspinosus* [18]. The protein has been named LS-12 and registered under Uniprot ID:P80961. This protein is approximately 12 kDa and 128 amino acids in length. The N-terminal 20 residues comprise a signal peptide for extracellular presentation, and the amino acid sequence of the AFP active domain from 21-128 is Gln/Glu-rich (∼17%).

In this way, four types have been described to date; however, many antifreeze proteins that are not classified in the above types have been discovered, and the above classifications are not sufficient to classify all antifreeze proteins that have been discovered. A great variety of AFPs have been found in insects, microorganisms, and fungi. Despite their functional similarities, AFPs show great diversity in their structural and sequence characteristics among species. Therefore, it is difficult to predict AFPs only by homology search.

Machine learning has been used to identify AFPs from amino acid sequences. Kandaswamy et al. [19] developed an AFP prediction system called AFP-Pred, which used random forest (RF) as a classifier. They extracted AFP sequences using the Pfam database [20] and collected their homologous sequences, eliminating redundant sequences with more than 40% identity by CD-HIT [21]. As a result, 481 sequences were obtained and were used as the positive dataset for prediction. Amino acid frequency, dipeptide frequency, secondary structure information, and physicochemical properties in the protein sequences were used as features. The AFP prediction tools AFP_PSSM [22], AFP_pseAAC [23], TargetFreeze [24] used support vector machine (SVM) as a classifier. AFP_PSSM used the position-specific scoring matrix (PSSM) as a sequence feature; AFP_pseAAC improved prediction performance using pseudo amino acid composition (pseAAC), and TargetFreeze further improved prediction performance by using pseudo PSSM in addition to pseAAC. Yang et al. [25] developed a tool called AFP-ensemble which used RF as a classifier and used many sequence features, including amino acid composition (AAC), dipeptide composition (DC), physicochemical properties, PSSM, disorder information, and functional domain. They compared performance between different features with RFs and SVM as classifiers. One unique contribution of their work is that it incorporates the features related to protein disorder. They considered that the disorder regions may relate to ice-binding because the disorder regions are always rich in binding sites and carry important roles in regulating protein functions [25]. Their work showed that the disorder's contribution is the smallest among their physicochemical properties, whereas our work suggests that the disorder is one of the important features as described later. Khan et al. developed another AFP prediction system called RAFP-Pred by using RF [26]. RAFP-Pred divides a protein sequence into two sub-sequences, calculates AAC and DC of each sub-sequence, and combines the results to obtain features. AFP-LSE, developed by Usman et al., uses an autoencoder and deep neural network with the feature “composition of *k*-spaced amino acid pairs.” The performance results when using AFP-LSE were as follows: balanced accuracy = 0.903; Youden's Index = 0.81; and Matthews correlation coefficient (MCC) = 0.52; these results indicate that this method performs better than the existing methods [27]. We discuss the performance of our method and that of AFP-LSE in the Results section. Usman et al. also developed another prediction method, AFP-SRC [28], which uses amino acid composition and dipeptide composition as features and also applied principal component analysis (PCA) to reduce the dimensions of features. discussed the effect of PCA comprehensively. Alim et al. developed PoGB-pred [29]; this prediction model was gradient boosting and the author applied PCA to reduce the dimensions of features, AAC, DC, and pseudo AAC. Alim et al. also discussed the effect of dimension reduction via a comparison with the performance of RAFP-Pred [26].

Recent studies also focus on the characterization of essential features for identifying AFPs. Pratiwi et al. [30] developed an AFP prediction tool called CryoProtect, which uses RF as a classifier and uses AAC and DC as sequence features. They also analyzed the sequences of AFPs and showed that Cys, Ser, Trp, Gly, Asn, and Thr were characteristic residues of AFPs while Leu, Val, Glu, Ile, and Met were characteristic of non-AFPs. These results reflected the amino acid propensities of AFP and non-AFPs, and the Gini index from the RF model for evaluating and ranking the feature importance of amino acids was complementary to the above results. Eslami et al. [31] developed an AFP prediction tool called afpCOOL, which uses SVM as a classifier and uses four types of descriptors: hydropathy, physicochemical properties, AAC, and evolutionary profile (400 descriptors). They showed physicochemical descriptors are the most informative features to discriminate between AFPs and non-AFPs. Sun et al. [32] developed a tool for identifying antifreeze proteins which used SVM as a classifier and evolutional information derived from PSSM as sequence features. They showed interesting findings that Cys, Trp, and Gly are conservative, and their replacements by Ala, Met, and Ala, respectively, are rare in AFPs. In this work, we applied AAC, DC, the composition of two amino acids with one arbitrary amino acid in between, and composition, transition, and distribution (CTD) based on various amino acid properties as described in the “Materials and Methods” section. Thus a wide range of features that are essential for the identification of AFPs can be found.

As for the dataset of AFP used for the machine learning benchmark, the above AFP prediction tools used the AFP-Pred dataset [19] as the basis. However, this dataset is limited in number, and it contains some sequences that we could not find any evidence or annotation related to antifreeze function, such as many C-type lectins and MADS-box domain-containing protein. UniProtKB contains a large number of sequences annotated as AFP and related keywords. The collection of AFPs from UniProtKB should be reconsidered. For this reason, RAFP-Pred [26] used 41 sequences from Protein Data Bank (PDB) and 369 sequences from UniProtKB, and the sequences in the AFP-Pred dataset. Eslami et al. also used 517 AFP sequences from UniProtKB and the same number of non-AFP sequences using PDB structures. They showed the results of performance analysis for the AFP-Pred dataset and the UniProtKB-based dataset. In this work, we constructed datasets of AFPs considering UniProtKB's reliability and removed the redundancy based on sequence identities and organic species. The current annotated sequences of UniProtKB are biased and we tried to identify common features that are characteristic to AFPs regardless of sequence families and species.

The current study does not predict the ice-binding residues of AFP directly, as in the study of Yu et al. [33] who used the PDB structures as a positive dataset for the purpose of predicting ice-binding sites. Although the dataset was limited and the accuracy of AFP identification was evaluated using this limited dataset, the identification of ice-binding sites was unique. In the present study, the amino acid motifs that ranked high in terms of feature score (see the Results) can be regarded as candidates for functional sites (not limited to ice-binding sites) of AFP.

**Figure 1:**
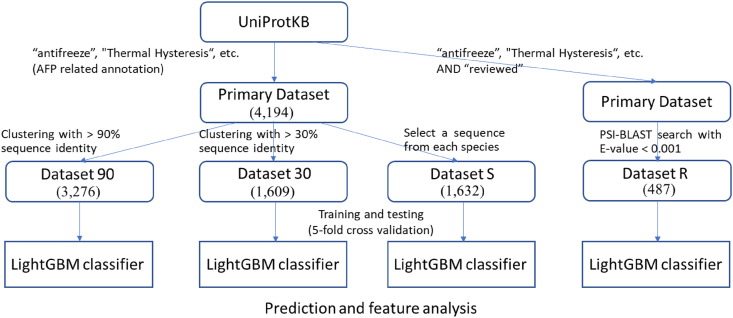
Outline of the present research. The numbers in parentheses represent the number of sequences in each dataset.

Figure 1 illustrates the outline of this work. We first constructed datasets of AFPs considering reliability, developed a binary classification prediction system based on these datasets, and discussed the features of AFPs that contributed to the prediction.

## 2 Materials and methods

### 2.1 Datasets

We first searched UniProtKB for AFP sequences using “antifreeze” and the synonyms of “antifreeze proteins” registered in NCBI MeSH as keywords. InterPro and PRINTS, linked to UniProtKB, were also specified as search targets. The total number of AFP sequences collected was 4,194. The details of the collection method (search keywords) are described in Table S1. We call the dataset of these sequences the primary dataset.

Using the primary dataset, we constructed the non-redundant datasets: Dataset 30 and Dataset 90 created by clustering the primary dataset with 30% and 90% sequence identity cutoffs, respectively, using PSI-CD-HIT (version 4.5.3) [21]. In Dataset 30, sequences from a Pfam family were grouped into a single and several clusters for some families. For example, 279 sequences with the SAF domain (PF08666) were divided into six clusters; 69 sequences with the AFP domain (PF02420) were grouped into one cluster; 32 sequences with the CfAFP domain (PF05264) were divided into four clusters; 244 sequences with NeuB domain (PF03102) were divided into six clusters. Dataset 30 and Dataset 90 consists of 1,609 and 3,276 sequences, respectively.

**Figure 2:**
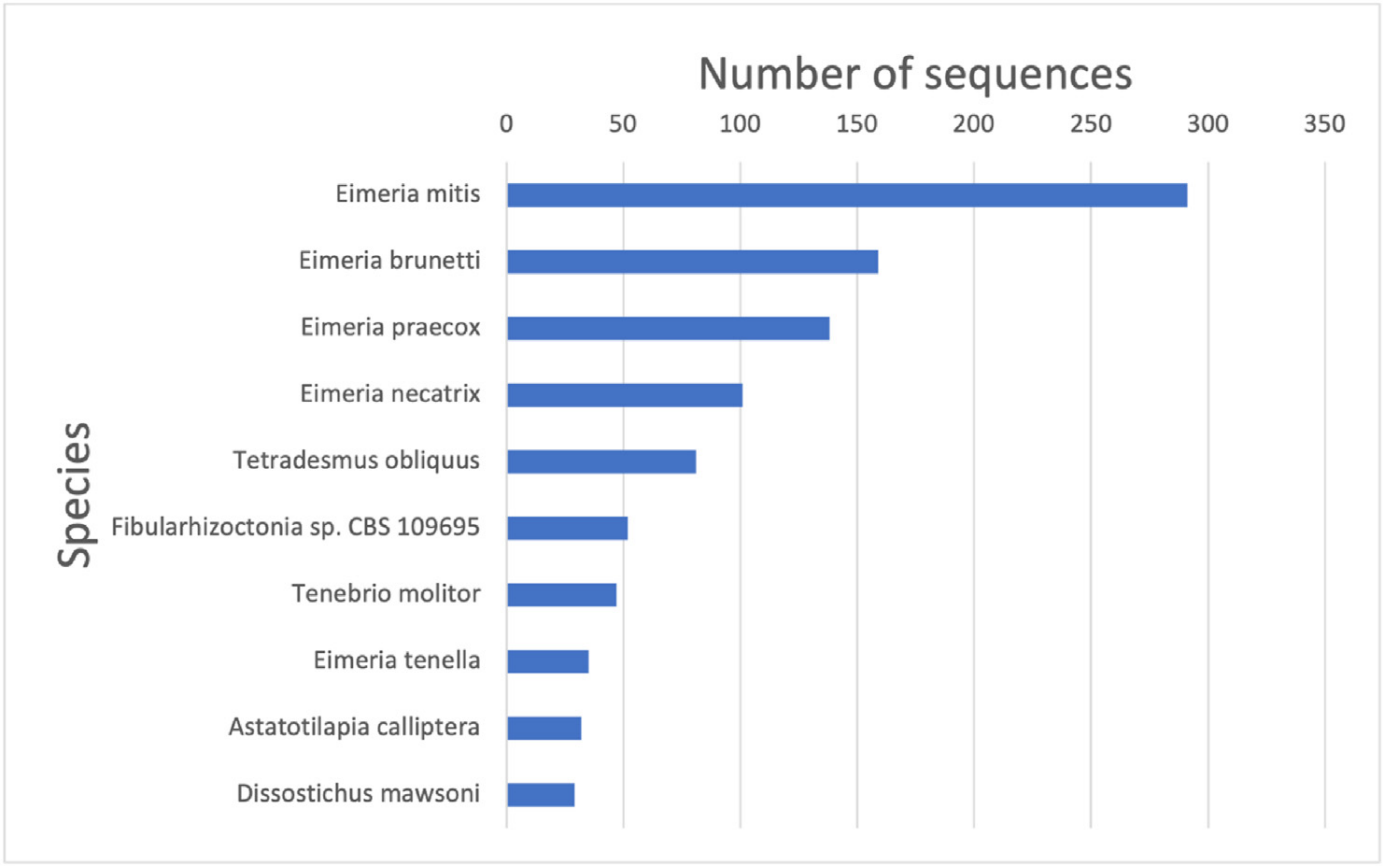
Top 10 species of the primary dataset.

The sequences in the primary dataset were derived from 1,632 species. Figure 2 shows the species with the ten largest number of sequences along with their dataset. There is a certain amount of bias by species. To eliminate the bias's possible influence, we also constructed another dataset, Dataset S, by selecting a representative sequence from each species. Dataset S consists of 1,632 sequences.

Among the 4,194 sequences in the primary dataset, there were 57 sequences with “reviewed” annotations of UniProtKB, which means that the sequence is manually annotated and considered to be highly reliable. Other sequences are “unreviewed,” which contains computationally generated annotations and protein sequences from large-scale functional analysis experiments. We performed the PSI-BLAST search (version 2.9.0+) with E-value < 0.001 for the 57 sequence. The number of hit sequences was 29,255. Out of 4,137 “unreviewed” sequences in the primary dataset, 430 sequences were included in the hit sequences, and they were added to the “reviewed” 57 sequences to construct Dataset R. The number of sequences in the Dataset R is 487.

Besides these datasets, we also prepared the dataset used in AFP-Pred. This dataset was collected based on the Pfam database; 221 sequences were collected from the Pfam database, and homologous sequences were collected with a strict threshold (E-value 0.001) by PSI-BLAST search. The final dataset contained only protein sequences with less than 40% identity after redundancy elimination using CD-HIT. We call this dataset Dataset AP. Dataset AP consists of 481 sequences.

**Figure 3:**
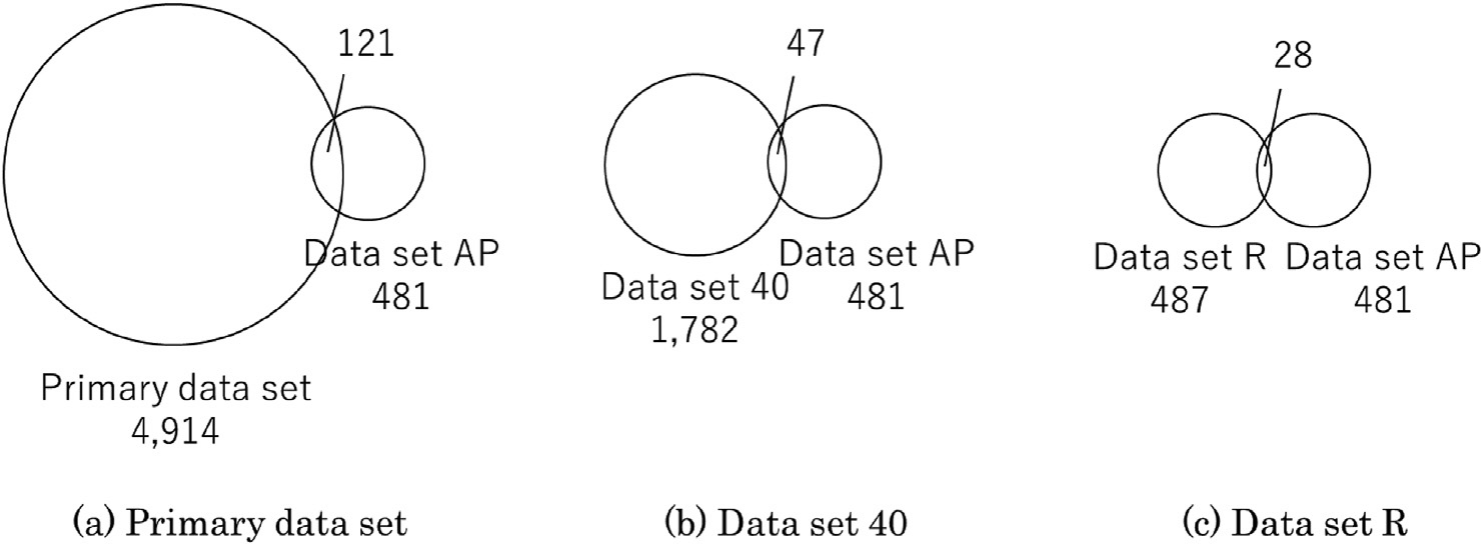
Comparison between Dataset AP and other datasets.

Figure 3 (a) shows the comparison of Dataset AP and the primary dataset. As shown in Figure 3 (a), 121 sequences are included in common. To compare the sequences between Dataset AP and the primary dataset at the same redundancy level, we created Dataset 40 by removing redundancy in the primary dataset with a 40% identity cutoff using PSI-CD-HIT. The number of sequences in Dataset 40 is 1,782, and the number of sequences common with Dataset AP is 47, as shown in Figure 3 (b). For the other redundancy levels, Dataset 90 and Dataset 30 have 109 and 38 sequences common to Dataset AP, respectively. Figure 3 (c) compares Dataset AP and Dataset R. The number of common sequences is 28. These figures show that the Dataset AP includes a tiny part of AFP-annotated sequences in UniProtKB.

The above datasets, Dataset 90, Dataset 30, Dataset S, and Dataset R, were used as positive datasets of machine learning. We constructed a non-AFP dataset (negative dataset) by randomly selecting the sequences tagged as “reviewed” from UniProtKB, excluding those hit by the search under the keywords “antifreeze” and “afp.” The resulting number of sequences in the negative dataset was 560,147; however, for training and testing, the same number of sequences was selected from the negative dataset as were selected from the positive dataset. Therefore, all training was performed on balanced positive and negative datasets. Table 1 summarizes the number of sequences of each dataset.

**Table 1:** Number of sequences in the datasets.

Datasets		Number of sequences
Primary dataset	Sequences annotated with AFP-related keywords	4,194
Dataset 90	Non-redundant sequences <90% sequence identity	3,276
Dataset 40	Non-redundant sequences <40% sequence identity	1,782
Dataset 30	Non-redundant sequences <30% sequence identity	1,609
Dataset S	Dataset of the representative sequence for each species	1,632
Dataset R	Reviewed sequences plus their 90% similar sequences	487
Dataset AP	Dataset used in AFP-Pred	481
Negative dataset^+^	UniProtKB sequences excluding AFP-related keywords. For training, the same number of negative sequences was selected from this dataset as were selected from the positive datasets.	560,147

Dataset 40 is only used for comparison with Dataset AP (same redundancy level).

### 2.2 Features

In this work, we used the following features for machine learning:

1. Amino acid (AA) composition (AAC).
2. Dipeptide composition (DC).
3. Composition of two amino acids with one arbitrary amino acid in between (AA-x-AA composition).
4. CTD.

The AAC can be expressed as a 20-dimensional vector. The DC is a composition of two consecutive amino acids, expressed as a vector of 20 × 20 = 400 dimensions. The AA-x-AA composition is a composition of the pattern AA-x-AA where x is an arbitrary amino acid and can also de expressed as a vector of 20 × 20 = 400 dimensions. This feature was used because some AFPs have this sequence pattern, especially in the ice-binding regions [34, 35]. CTD is a feature that indicates the distribution of amino acid patterns along the primary sequence of a protein, based on physicochemical and structural properties [36, 37]. C (Composition) indicates the composition of amino acids of a particular property; T (Transition) indicates the percent frequency with which amino acids with a different property follow amino acids with a particular property; *D* (Distribution) indicates the distribution of the initial, 25%, 50%, 75%, and 100% positions of the amino acids with a particular property.

In this work, eight properties: hydrophobicity, normalized van der Waals volume, polarity, charge, secondary structure, solvent accessibility, polarizability, and disorder propensity [38], were considered as shown in Table S2. Each amino acid was classified into three classes Group 1, 2, and 3 for each property. For each property, Composition has a three-dimensional feature, Transition has a three-dimensional feature, and Distribution has a 15-dimensional feature (three groups times five positions). Therefore, the dimension of the CTD features of this work is 8×(3+3+15)=168. As an example, consider the sequence “MAGGDLVYAGSIAEHRKL.” When we consider the polarizability of this sequence, it can be encoded as “311112231112123332” according to the grouping described in Table S2. Figure S1 shows the detailed calculation of CTD for this example. The compositions of group 1, group 2, and group 3 are 8/18, 5/18, and 5/18, respectively. The compositions of transition 1/2, transition 1/3, and transition 2/3 are 4/17, 2/17, and 3/17, respectively. Transition a/b represents transition a to b or transition b to a. The distributions of the initial (first), 25% (second), 50% (fourth), 75% (sixth), and 100% (eight) of group 1 were calculated as 2/18, 3/18, 5/18, 10/18, and 13/18, respectively.

### 2.3 Classification method

We used LightGBM [39] as a machine learning framework. LightGBM is a type of Gradient Boosting Decision Tree (GBDT) and uses an ensemble learning method that combines several weak learners (decision trees) to build a single strong learner. LightGBM is particularly unique among GBDTs in that it employs the Leaf-wise method, Gradient-based One-Side Sampling (GOSS), and Exclusive Feature Bundling (EFB). Figure S2 shows the flowchart of LigthtGBM.

The Leaf-wise method is used in the decision tree construction process of the GBDT algorithm [40]. In LightGBM, Leaf-wise is used because it has an advantage in short training time. GOSS is a learning method that excludes data with small gradients and samples only data with large gradients [41]. GOSS's advantage is that the computational cost can be significantly reduced because the instance split point is determined using the estimated value of variance gains. On the other hand, EFB is a method to reduce the number of features by bundling mutually exclusive features. This is based on an idea that the computational cost of searching for branch points is so heavy that it is not necessary to search for the entire features. Especially in large datasets, there are many sparse features, and it is not uncommon for the features of non-zero elements to have no overlap at all, such as one-hot encoding features.

### 2.4 Evaluation method

We performed five-fold cross-validation on each dataset. Five-fold cross-validation divides the dataset into five blocks and treats one data block as the test data and the remaining data blocks as the training data. By repeating this process five times, each divided data block is treated as the test data.

### 2.5 Performance measure

The precision, specificity, recall, accuracy, and MCC were used to evaluate the prediction performance in this work. These are expressed as follows:

$$Precision=\frac{TP}{TP\;+FP}$$

$$Specifity=\;\frac{TN}{TN\;+\;FP}$$

$$Recall=\frac{TP}{TP\;+FN}$$

$$Accuracy=\frac{TP\;+TN}{TP\;+FP\;+TN\;+FN}$$

$$MCC=\frac{TP・TN\;-FP・FN}{\sqrt{\left(TP\;+FP\right)\left(TP\;+FN\right)\left(TN\;+FP\right)\left(TN\;+FN\right)}}$$

where TP, FP, TN, and FN are: true positive, false positive, true negative, and false negative, respectively. Precision is the percentage of true positive sequences against the sequences that are predicted to be positive, specificity is the percentage of true negative sequences against the truly negative sequences, recall is the percentage of true positive sequences against sequences that are truly positive, and accuracy is the percentage of the correctly predicted sequences against the total sequences. MCC takes account of the accuracy of prediction for both positive and negative sequences.

The Receiver Operating Characteristic curve (ROC curve) was also used to visualize the prediction performance. The ROC curve is a plot of the false positive rate (*= FP*/(*TN + FP)*) against the true positive rate (= reproducibility; recall).

## 3 Results and discussions

### 3.1 Prediction performance

**Figure 4:**
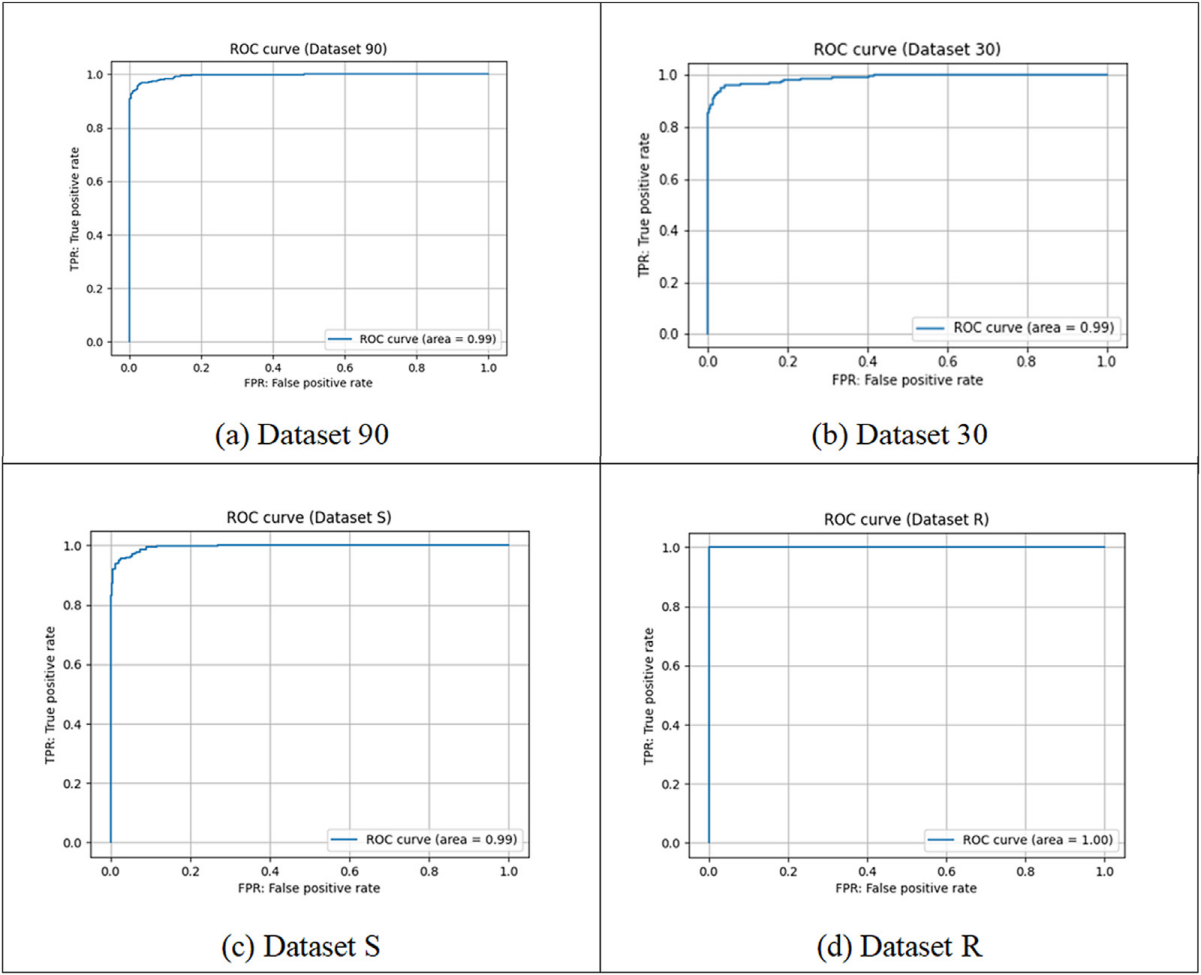
ROC curves.

The prediction performances for the four datasets are shown in Table 2. The performance was evaluated based on the accuracy (ACC), specificity (SPE), recall (REC), and MCC using five-fold cross-validation. The average value of each performance measure in the five-fold cross-validation is shown in Table 2. LightGBM created a classification predictor for each dataset. The LightGBM Tuner was used to find optimal hyperparameter values of the model. Figure 4 (a)-(d) shows the ROC curves for Dataset 90, Dataset 30, Dataset S, and Dataset R, respectively.

**Table 2:** Prediction performance.

	Accuracy	Precision	Recall	MCC	AUC	Youden's index
Dataset 90	0.961	0.978	0.944	0.923	0.994	0.919
Dataset 30	0.947	0.979	0.914	0.896	0.989	0.882
Dataset S	0.953	0.973	0.934	0.908	0.994	0.990
Dataset R	0.986	0.992	0.979	0.972	1	0.945

Dataset 90 has a larger number of sequences and facilitates learning compared with Dataset 30. Many sequences of specific species (*e.g., Eimeria*) are included in the primary dataset (Dataset 90 and Dataset 30), which may influence the precision of AFP of the specific species to some extent. Accuracy, recall, and MCC of Dataset S are higher than Dataset 30. Although Dataset S only contains one sequence for each species, sequence redundancy was not removed. If some common sequence properties exist in AFPs among species, the learning effect seems higher than Dataset 30. Since Dataset R consists of 57 “reviewed” sequences and their close homologs, there may be a possibility that learning and testing were performed within a limited variety of sequences.

We performed independent tests on Dataset S and Dataset R. In both datasets, 64% of the whole dataset was treated as the training set, 16% as the validation set, and 20% as the test set. The prediction performances are shown in Table 3. Good performance was also obtained via an independent test.

**Table 3:** Prediction performances of independent tests.

	Accuracy	Precision	Recall	MCC	AUC	Youden's index
Dataset S	0.946	0.958	0.930	0.893	0.986	0.900
Dataset R	0.990	0.981	1	0.980	0.999	1

We also performed three cross-data tests by training the model on Dataset 90, Dataset 30, and Dataset S (as well as each of the negative datasets) and by testing it on Dataset R. Table 4 shows the performance of these cross tests.

**Table 4:** Prediction performance of cross testing.

	Accuracy	Precision	Recall	MCC	AUC	Youden's index
Training on Dataset 90 and Testing on Dataset R	0.990	0.990	0.990	0.979	1	0.986
Training on Dataset 30 and Testing on Dataset R	0.729	0.991	0.462	0.542	0.912	0.688
Training on Dataset S and Testing on Dataset R	0.982	0.990	0.973	0.963	0.999	0.971

Results from cross-testing Dataset 90 and Dataset R showed high performance. In this case, the features of AFPs seemed to be trained and used for predicting AFPs. However, the performance from cross-testing Dataset 30 and Dataset R was relatively low. This suggests that there are some features of AFPs that failed to be trained by removing the sequence redundancy. Cross-testing Dataset S and Dataset R also resulted in a high performance, although the number of sequences in Dataset 30 and Dataset S are almost the same. Since Dataset S consists of representative sequences for each species, the features common to the AFPs of each species were effectively trained. In other words, the features with high scores in Dataset S are considered to be included in features common to the AFPs of each species.

For comparison with other methods, we evaluated the performance of a recent method, AFP-LSE [27], relative to our method. Independence tests on Dataset S and Dataset R were performed under the same conditions: 64% of the whole dataset was treated as the training set, 16% as the validation set, and 20% as the test set. The prediction performances are shown in Table 5. Comparing the results from the two methods, our method outperformed AFP-LSE for both Dataset S and Dataset R (Table 3). Since AFP-LSE was based on the AFP-Pred dataset [19], it could not train the features of our datasets that were collected from a broader set of AFP sequences.

**Table 5:** Performance comparison of our method with AFP-LSE.

	Accuracy	Precision	Recall	MCC	AUC	Youden's index
Data set S	0.896	0.897	0.886	0.791	0.950	0.795
Data set R	0.841	0.929	0.760	0.698	0.950	0.812

### 3.2 Contributing features to the prediction

From the features that contributed to the classification prediction, it is possible to investigate the antifreeze function's essential properties. Table 6 (a)-(d) shows the top ten features with a considerable contribution to classification. In particular, the result of Dataset 90 (a) shows the overall tendency of AFPs, the result of Dataset 30 (b) shows some properties common to domains and families of AFPs, the result of Dataset S (c) shows the properties common across different species in biological taxonomy, and the result of Dataset R (d) shows the tendency of the current, reliable AFP sequences.

**Table 6:** Top 10 features contributing to classification prediction.

	Feature	Score
**a) Dataset 90**		
1	Polarity Composition; Group 2	1.57 × 10^−1^
2	Cys	7.81 × 10^−2^
3	Ala-Ala	6.69 × 10^−2^
4	Ala-x-Ala	4.50 × 10^−2^
5	Disorder Propensity Distribution; Group 1 (50%)	3.27 × 10^−2^
6	Met-Gly	3.24 × 10^−2^
7	Trp-x-Asp	2.57 × 10^−2^
8	Normalized VDWV Composition; Group 1	2.42 × 10^−2^
9	Trp-Gly	1.89 × 10^−2^
10	Hydrophobicity Composition; Group 2	1.47 × 10^−2^
**b) Dataset 30**		
1	Ala-x-Ala	2.73 × 10^−1^
2	Ala-Ala	2.65 × 10^−1^
3	Cys	4.33 × 10^−2^
4	Polarity Composition; Group 2	3.20 × 10^−2^
5	Ala	2.57 × 10^−2^
6	Polarity Composition; Group 1	1.56 × 10^−2^
7	Normalized VDWV Composition; Group 1	1.41 × 10^−2^
8	Leu	1.13 × 10^−2^
9	Polarizability Composition; Group 1	7.72 × 10^−3^
10	Hydrophobicity Composition; Group 2	6.68 × 10^−3^
**c) Dataset S**		
1	Disorder Propensity Distribution; Group 2 (50%)	8.68 × 10^−2^
2	Disorder Propensity Distribution; Group 1 (50%)	5.24 × 10^−2^
3	Ala	4.39 × 10^−2^
4	Polarity Composition; Group 2	3.86 × 10^−2^
5	Cys	3.73 × 10^−2^
6	Trp-Gly	2.93 × 10^−2^
7	Normalized VDWV Composition; Group 1	2.90 × 10^−2^
8	Hydrophobicity Composition; Group 2	1.97 × 10^−2^
9	Ala-Ala	1.96 × 10^−2^
10	Arg	1.92 × 10^−2^
**d) Dataset R**		
1	Disorder Propensity Distribution; Group 3 (50%)	6.18 × 10^−2^
2	Polarity Composition; Group 2	5.59 × 10^−2^
3	Trp-Gly	4.89 × 10^−2^
4	Normalized VDWV Composition; Group 1	4.57 × 10^−2^
5	Cys	3.82 × 10^−2^
6	Arg	3.19 × 10^−2^
7	Ala-Ala	2.24 × 10^−2^
8	Ala	2.15 × 10^−2^
9	Disorder Propensity Distribution; Group 1 (50%)	1.63 × 10^−2^
10	Leu-Leu	1.59 × 10^−2^

Score refers to a normalized decrease (Gini impurity) in the objective function from the decision branch that uses the feature. Groups 1–3 are classifications of AA properties shown in Table S1.

Many of the features that contributed to prediction in Dataset 30 are similar for prediction in Dataset 90; this shows that these features are shared among the various families and AFP domains. Focusing on the AA characteristics, Cys content, Ala-Ala content, Ala-x-Ala content, Met-Gly content, Trp-x-Asp content, and Trp-Gly content of AFP are much larger than those of other proteins. The Cys content is higher in Type-2 AFP, and the Ala-Ala and Ala-x-Ala contents are higher in Type-1 AFP. In particular, Ala-Ala and Ala-x-Ala are the most abundant peptides on the ice-binding surface of Type-1 AFP [42]. Met-Gly, Trp-Xx-Asp, and Trp-Gly are also considered essential peptides on the ice-binding surface of AFP physicochemical properties; AFP contains more AA with moderate polarity, small van der Waals volume (VDWV), and moderate hydrophobicity than other proteins. As for the VDWV, it is thought that the AA with a large VDWV can sterically inhibit other AAs from binding to ice through van der Waals interactions and hydrogen bonds.

As shown in Table 2 (c), the contribution of high (Group 1) and moderate (Group 2) disorder propensities is high compared with the above datasets. This shows disorder seems to have some relationship with antifreeze function regardless of species, although such a relationship has not yet been reported.

The features such as the Cys content, Ala-Ala content, Trp-Gly, polarity composition, small van der Waals, moderate hydrophobicity also appear as common features. On the other hand, Met-Gly content and Trp-x-Asp content, which contributed to Dataset 90 but did not contribute significantly to the prediction for the other datasets, might be characteristic of the specific group of sequences.

## 4 Conclusion

In this work, we created a new dataset of AFPs, made binary classification predictions for AFPs and non-AFPs, and discussed the properties of AFPs based on the features that contributed to the classification predictions.

As described in the “Datasets” section, the reliability of the sequences in the dataset used in AFP-Pred (Dataset AP) is not high, and therefore, in this work, we carefully collected the AFP sequences. The AFP sequences in the primary dataset (selected from UniProtKB) contain many sequences from specific species, as shown in Table 1. Dataset S was constructed by eliminating the influence of any bias of the species.

The classification predictors developed in this work showed high accuracy, precision, and recall. They can be used as tools for predicting whether or not a given protein is an AFP in proteome-wide analyses. As shown in the results for non-redundant datasets, AFP prediction by sequence homology (similarity) seems to be difficult. However, machine learning was a useful technique for learning the common features of AFPs.

Regarding the features that contributed to the prediction of AFP and non-AFP classification, several features contributed commonly. A typical example is short AA sequence motifs, including Ala, Cys, Trp, and Gly. Our results also suggest that the disorder propensity was related to the function of AFPs. For further research, it is necessary to confirm the antifreeze property by experimental methods.

A small number of reliable annotated AFP sequences (Dataset R) exist in the current database. In the present study, we obtained a high AUC value of 0.99 in the independent test of Dataset R. However, a lower AUC value of 0.767 was obtained by training with Dataset R and performing a cross test with Dataset S. This may be due to the small number of sequences in Dataset R and the characteristics of AFP not being fully learned, despite the potential inclusion of sequences other than AFP in Dataset S. To develop a predictor that is more reliable, it will be desirable to achieve reliable annotations of AFP sequences.

As future research to extend the present study, it will be necessary to confirm thermal hysteresis activity via experimental methods. For example, by mutating amino acid residues with high disorder tendencies using those with low disorder tendencies, and then by comparing the thermal hysteresis activity, the relationship between the disorder tendencies of amino acids and the function of AFP could be further elucidated.

## Declarations

### Author contribution statement

Ryosuke Miyata: Conceived and designed the experiments; Performed the experiments; Analyzed and interpreted the data; Contributed reagents, materials, analysis tools or data; Wrote the paper.

Yoshitaka Moriwaki: Analyzed and interpreted the data; Contributed reagents, materials, analysis tools or data.

Tohru Terada: Analyzed and interpreted the data; Contributed reagents, materials, analysis tools or data.

Kentaro Shimizu: Conceived and designed the experiments; Analyzed and interpreted the data; Contributed reagents, materials, analysis tools or data; Wrote the paper.

### Funding statement

This work was supported by Platform Project for Supporting Drug Discovery and Life Science Research (Basis for Supporting Innovative Drug Discovery and Life Science Research (BINDS)) from 10.13039/100009619AMED under Grant Number JP21am0101107, and 10.13039/100008732The Uehara Memorial Foundation under Grant Number 202080071.

### Data availability statement

Data associated with this study has been deposited at https://github.com/ryomiya/Prediction-and-analysis-of-antifreeze-proteins.

### Declaration of interests statement

The authors declare no conflict of interest.

### Additional information

No additional information is available for this paper.

## Appendix A Supplementary data

Supplementary information_V2 Supplementary information_V2
