# Supplementary material for: Prediction and analysis of antifreeze proteins
Source: Heliyon. 2021 Sep 8;7(9):e07953. doi: 10.1016/j.heliyon.2021.e07953 (PMC8473546; doi:10.1016/j.heliyon.2021.e07953)
Supplement: Supplementary information_V2 [file mmc1.docx]

**Supplementary information**

**Table S1** AFP sequence search in UniProtKB (https://www.uniprot.org/)

| Search word list | Search target item |
| --- | --- |
| antifreeze  “Thermal Hysteresis.”  "Hysteresis Proteins, Thermal.”  AFGP  “Fish Type III AFP.”  “Fish Type II AFP”  “Fish Type I APF.”  “Fish Type IV AFP.”  “Carrot AFP protein, Daucus carota.”  “sea raven AFP” | name  annotation:(type:biophysicochemical_properties)  annotation:(type:function)  annotation:(type:positional)  annotation:(type: “positional domain”)  annotation:(type: “non-positional domain”)  family  goa  keyword |
| antifreeze_1  insect_antifreeze_prot_motif  antifreeze_iii  antifreeze_cf  insect_cyst_antifreeze_prot  antifreeze_sf | database: (type:interpro) |
| antifreezei  antifreezeii  antifreezeiii | database:(type:prints) |

**Table S2** Amino acid properties and grouping in CTD.

| Property | Group 1 | Group 2 | Group 3 |
| --- | --- | --- | --- |
| Hydrophobicity | Polar | Neutral | Hydrophobicity |
| Amino acids | D,E,K,N,Q,R | A,G,H,P,S,T,Y | C,F,I,L,M,V,W |
| Normalized VDWV | 0–2.78 | 2.95–4.0 | 4.03–8.08 |
| Amino acids | A,C,D,G,P,S,T | E,I,L,N,Q,V | F,H,K,M,R,W,Y |
| Polarity | 4.9–6.2 | 8.0–9.2 | 10.4–13.0 |
| Amino acids | C,F,I,L,M,V,W,Y | A,G,P,S,T | D,E,H,K,N,Q,R |
| Charge | Positively Charged | Neutral | Negatively Charged |
| Amino acids | K,R | A,C,F,G,H,I,L,M,N,P,Q,S,T,V,W,  Y | D,E |
| Secondary Str | Helix | Strand | Coil |
| Amino acids | A,E,H,K,L,M,Q,R | C,F,I,T,V,W,Y | D,G,N,P,S |
| Solvent accessibility | Buried | Exposed | Intermediate |
| Amino acids | A,C,F,G,I,L,V,W | D,E,K,N,R,Q | H,M,P,S,T,Y |
| Polarizability | 0–0.108 | 0.128–0.186 | 0.219–0.409 |
| Amino acids | G,A,S,D,T | C,P,N,V,E,Q,I,L | K,M,H,F,R,Y,W |
| Disorder Propensity[38] | Disorder residue | Promoting residue | Neutral |
| Amino acids | A,R,S,Q,E,G,K,P | I,L,N,C,F,Y,V,W | D,H,M,T |


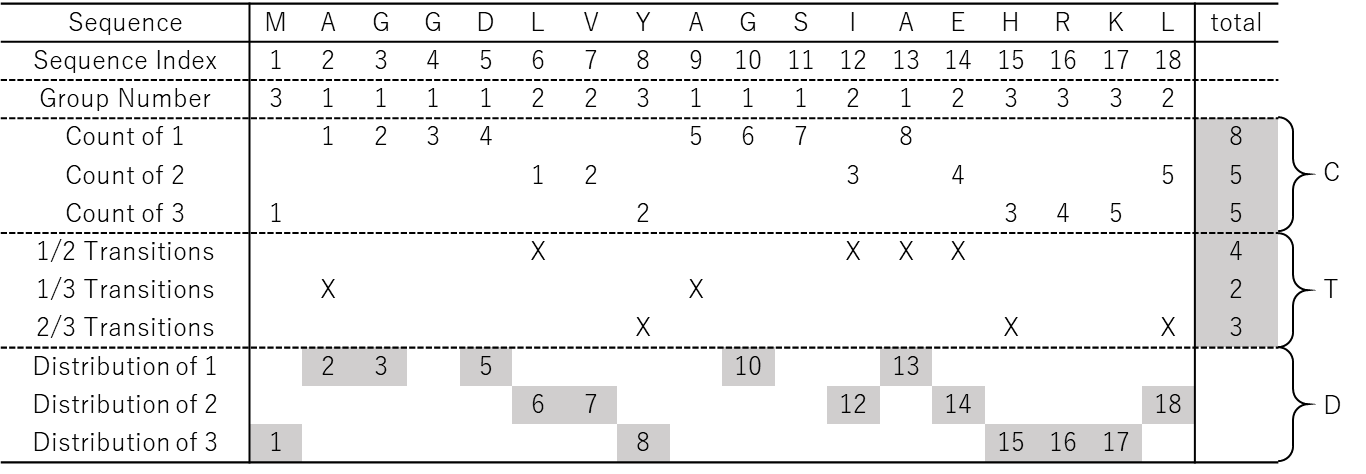


**Figure S1** Calculation of CDT

Calculation of CDT for the amino acid sequence “MAGGDLVYAGSIAEHRKL.” The sequence is encoded as “311112231112123332” according to the grouping of polarizability described in **Table S2.** Let *n* be the length of the sequence (*n* = 18 in this case). Composition *C* is calculated by dividing the total by *n*. Transition *T* is calculated by dividing the total by *n −* 1. Distribution *D* is calculated by diving each number by *n*.

**
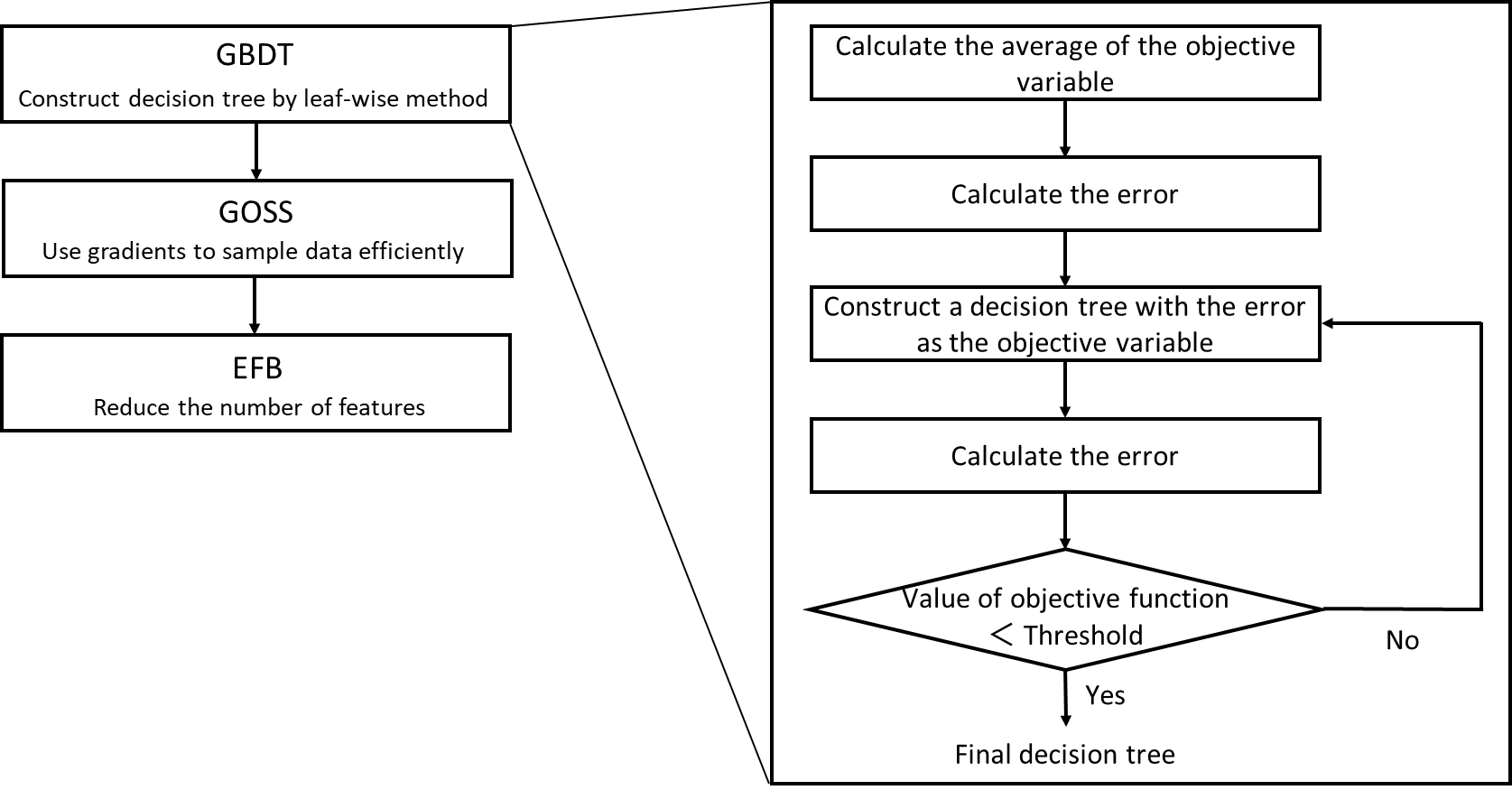
**

**Figure S2 Flowchart of LightGBM process.**
